# Renal failure due to encrusted cystitis and pyelitis

**DOI:** 10.1002/iju5.12158

**Published:** 2020-04-17

**Authors:** Katsuhiro Ito, Toshifumi Takahashi, Toru Kanno, Takashi Okada, Yoshihito Higashi, Hitoshi Yamada

**Affiliations:** ^1^ Department of Urology Ijinkai Takeda General Hospital Kyoto Japan

**Keywords:** *Corynebacterium urealyticum*, encrusted cystitis, encrusted pyelitis, infection, renal failure

## Abstract

**Introduction:**

Encrusted cystitis and pyelitis are a rare urinary tract infection characterized by mold‐like calcification of collecting system. Here, we show a case of encrusted cystitis proceeding to pyelitis during a 1‐month delay in diagnosis.

**Case presentation:**

A 73‐year‐old man developed hematuria and pain during micturition while he was being treated for granulomatosis with polyangiitis and lung abscess. Cystoscopy revealed calcification of the bladder wall, and an initial diagnosis of a bladder stone was made. While awaiting surgery, the bladder wall calcification extended to the renal pelvis on both sides, with renal failure. He underwent bilateral nephrostomy replacement and bladder irrigation with Solita T1 and was administered intravenous vancomycin. Calcification almost regressed after 4 weeks of treatment.

**Conclusion:**

Encrusted cystitis and pyelitis should be suspected if the patient shows alkaline urine and urothelial mucosa calcification. Appropriate treatment includes antibiotics, urine drainage, and chemolysis by bladder irrigation.

**Abbreviations & Acronyms**
CRP: C‐reactive protein
CT: computed tomography
EC: encrusted cystitis
EP: encrusted pyelitis
PNS: percutaneous nephrostomy
PSL: prednisolone
TUCL: transurethral cystolithotripsy
WBC: white blood cell

**Keynote message**

We report a case of EC proceeding to pyelitis with renal failure during a 1‐month delay in diagnosis. Early suspicion of the disease is necessary if the patient shows alkaline urine and urothelial mucosa calcification.

## Introduction

EC and EP are a rare urinary tract infection characterized by mold‐like calcification of the urinary collecting system.^1^ An immunosuppressed condition, use of broad‐spectrum antibiotics, and previous urological procedures are risk factors for EC and EP.^2^, ^3^ EC and EP are usually caused by urea‐splitting bacteria, mostly *Corynebacterium urealyticum*. This slow‐growing, drug‐resistant, gram‐positive bacterium is a commensal skin organism and is either missed in routine urine culture because it takes a long incubation time to grow well or considered contamination.^3^ Because of the rarity of EC and EP and difficulty in culture, diagnosis is sometimes substantially delayed, which could be critical. The clinical course of EC and EP is still sparsely described. Here, we present a case of EC that progressed to EP during a 1‐month delay in diagnosis.

## Case presentation

**Fig. 1:**
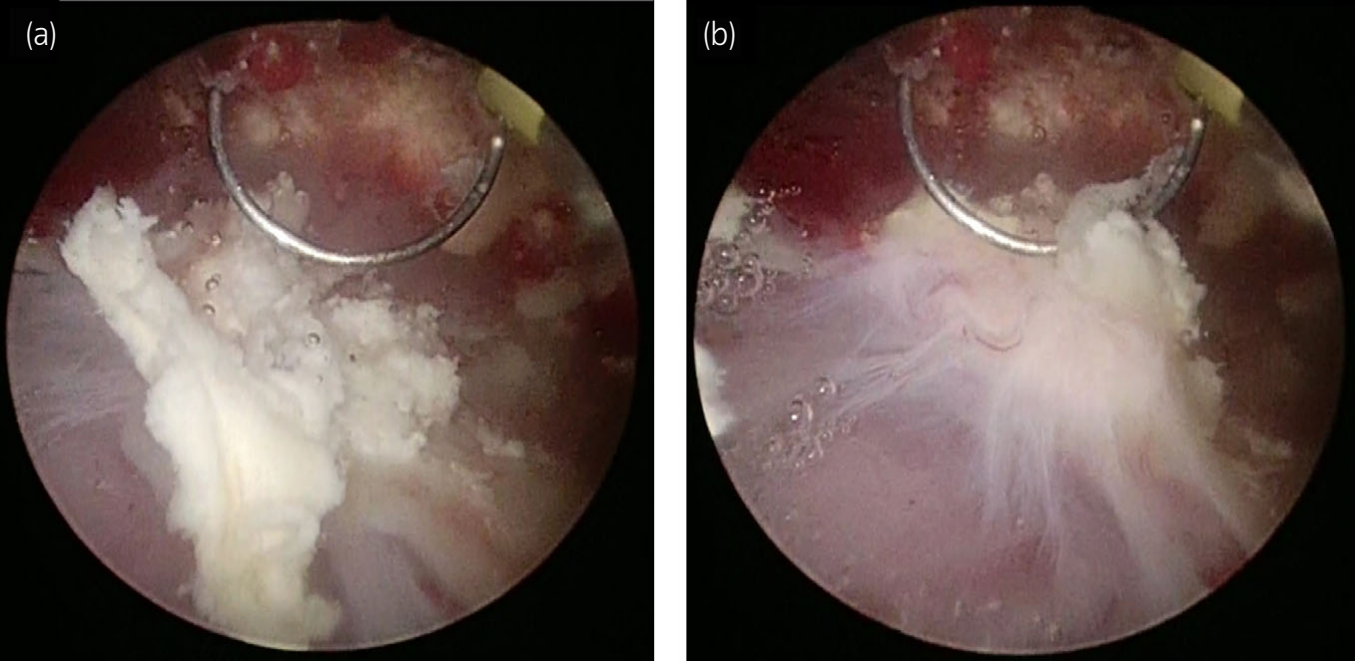
Endoscopic view of the patient’s EC.

**Fig. 2:**
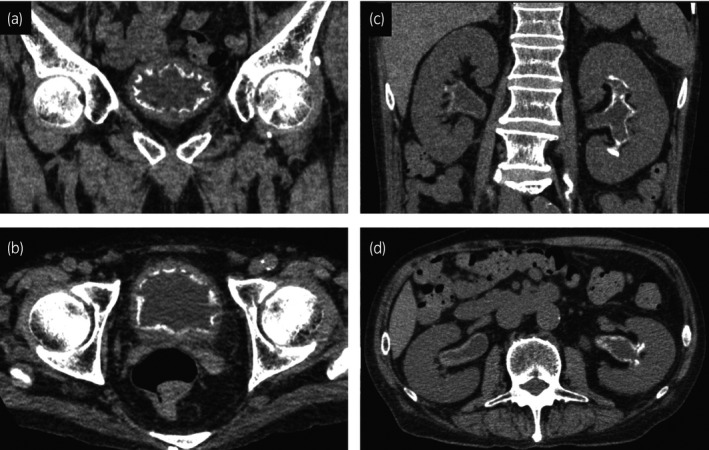
Coronal and axial CT images of bladder (a,b) and kidney (c,d). A thin layer of calcification along the bladder and pelvic wall was observed.

**Fig. 3:**
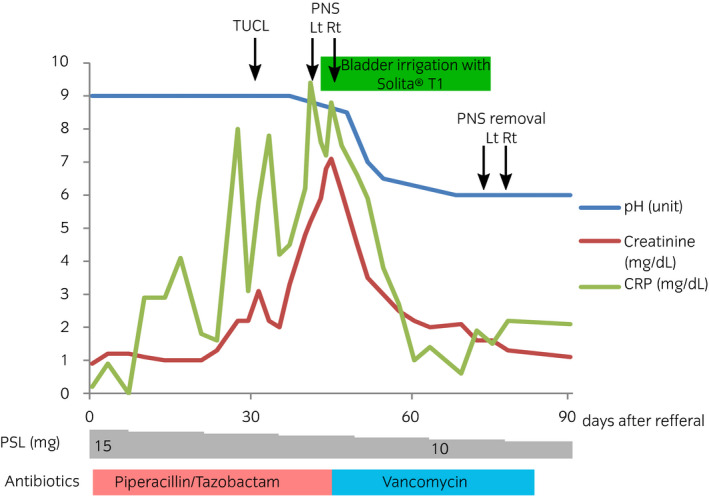
Clinical course of the patient.

A 73‐year‐old man developed hematuria and pain during micturition while he was being treated for granulomatosis with polyangiitis. He was administered oral PSL for 2 months at an initial dose of 30 mg, which was then gradually tapered to 15 mg. For 2 weeks, he was also being treated with 4.5 g of intravenous piperacillin/tazobactam every 12 h for lung abscess. His physical examination findings were normal. Laboratory results showed an increased WBC count of 14 600/μL, slightly increased CRP levels (0.9 mg/dL), and increased serum creatinine levels (1.2 mg/dL from 0.7 mg/dL). Urinalysis showed pyuria (>100 WBC/high‐power field) and alkaline urine (pH >9.0 units). Urine gram staining showed gram‐positive bacteria, but urine culture was negative. Cystoscopy showed multiple calcified debris clung to the bladder wall. CT scan showed slight calcification at the bottom of the bladder. One month after the initial visit, the patient underwent TUCL. The calculi were sticky and firmly adhered to the bladder mucosa (Fig. 1). They were only partially removed because the bladder mucosa easily peeled off and started to bleed. Stone analysis showed 67% struvite and 33% carbonate‐apatite. The patient’s creatinine levels gradually increased to 4.84 mg/dL. CT scan showed increased calcification of the bladder and calcified walls of the renal calyx, renal pelvis, and ureter on both sides, with hydronephrosis (Fig. 2a–d). At that time, the patient was diagnosed with EC and EP. He underwent bilateral nephrostomy. Although repeat urine culture was negative, the antibiotic was switched to vancomycin. His bladder was irrigated with Solita^®^ T No. 1 (90 mEq/L of Na, 70 mEq/L of Cl, 20 mEq/L of L‐lactate, and 13 g/dL of glucose; pH: 3.5–6.5) at 50 mL/h. Calcification of the bladder, renal pelvis, and ureters almost regressed after 4 weeks of treatment. His creatinine levels gradually decreased to 1.0 mg/dL but did not reach the initial level. His urine pH returned to normal. Treatment of granulomatosis with polyangiitis was continued, without recurrence of EC and EP at 3 months’ follow‐up. Figure 3 shows the clinical course.

## Discussion

The patient’s clinical course illustrates two important and interesting points. First, renal calcification, which was not observed at the initial visit, appeared after 1 month. Most EP cases were reported to have renal calcification at the initial visit. Only in two case reports, EC was initially misdiagnosed as bladder stone, and EP developed a few months later.^4^, ^5^ These findings suggest that EC has a slowly progressive course over several months. Mortality^4^, ^5^, ^6^ or loss of renal function^7^ due to a delay in diagnosis has been reported. If adequately diagnosed and treated, the prognosis is considered favorable.^8^ Images of EC or EP are highly characteristic and essential for diagnosis. CT scan without contrast media shows thin calcification outlining the bladder and the ureteropelvic wall.^9^ Since *C. urealyticum* is difficult to culture, negative urine culture cannot rule out EC and EP. Prolonged urine culture, culture of the removed stone, or DNA–polymerase chain reaction for will support the diagnosis.^3^, ^10^ Therefore, the clinician’s awareness of EC and EP is most important.

Second, calcification of the renal pelvis regressed without surgical removal of stones or irrigation of the upper urinary tract. As the infection improved, the urine pH decreased and the calcification seemed to dissolve spontaneously. This finding suggests appropriate infection control with antibiotics and drainage is the most important. Even without identification of a definitive species, empirical glycopeptide use is allowed because *C. urealyticum* is resistant to most of antibiotics but usually sensitive to glycopeptide.^3^ EC or EP is rarely caused by other urea‐splitting bacteria, such as *Proteus mirabilis*, *Corynebacterium glucuronolyticum* or *Arcanobacterium pyogenes*.^11^, ^12^, ^13^ In the case of hydronephrosis, drainage via a nephrostomy tube or ureteral stent should also be considered. In this case, improvement in the patient’s renal function was slow, even after insertion of a nephrostomy tube, probably because the entirely encrusted pelvicmucosa, not ureter obstruction, caused renal failure.

Other complementary treatments include urine acidification. Urease produced by bacteria hydrolyzes urea to ammonia and carbon dioxide, causing alkalization of urine.^14^ At high pH, precipitation of NH4^+^ with magnesium and phosphate is promoted to form struvite, and precipitation of carbonate with phosphate form carbonate‐apatite. Bladder irrigation with acid solutions is often performed to increase solubility of struvite and calcium phosphate.^2^ In this case, we used Solita^®^ T1 for convenience, whereas Suby’s solution G or Thomas C24 is commonly used.^2^ Irrigation of the renal pelvis via nephrostomy for EP has been reported,^15^ but it has low tolerability due to pain and a risk of urosepsis.^7^, ^16^

## Conclusions

EC and EP should be suspected early on if the patient shows alkaline urine and calcification of the urothelial mucosa. EC and EP can be cured with appropriate treatment, including antibiotics, urine drainage, and acidification by bladder irrigation.

## Conflict of interest

The authors declare no conflict of interest.
